# Lifestyle choices of Brazilian college students

**DOI:** 10.7717/peerj.9830

**Published:** 2020-10-07

**Authors:** Bianca G. Martins, João Marôco, Mauro V.G. Barros, Juliana A.D.B. Campos

**Affiliations:** 1 Department of Biological Sciences/School of Pharmaceutical Sciences, São Paulo State University—UNESP, Araraquara, São Paulo, Brazil; 2 William James Center for Research (WJCR), Instituto Universitário de Ciências Psicológicas, Sociais e da Vida—ISPA, Lisbon, Portugal; 3 Lifestyles and Health Research Group, Universidade de Pernambuco, Recife, Pernambuco, Brazil

**Keywords:** Lifestyle, Health promotion, Students, Psychometrics, Epidemiology

## Abstract

**Background:**

Lifestyle choices reflect the beliefs that individuals attribute to aspects of life. This construct can be assessed with the Individual Lifestyle Profile (PEVI) questionnaire, which measures elements of Nutrition, Physical Activity, Preventive Behaviors, Social Relationships and Stress Management.

**Objective:**

The objective of this study was to estimate the psychometric properties of the PEVI applied to a sample of Brazilian university students, identifying the prevalence of each lifestyle component according to participants’ age, sex, weight status, course area/field and economic stratum and to estimate the contribution of these characteristics on physical and psychological lifestyle.

**Methods:**

The PEVI data was analyzed by confirmatory factor analysis, using the indexes chi-square per degrees of freedom ratio (χ^2^/df), Comparative Fit Index (CFI), Tucker-Lewis Index (TLI) and Root Mean Square Error of Approximation (RMSEA). First-order and second-order models (physical and psychological lifestyle) were tested. Prevalences of lifestyle components were calculated and compared by participants’ age, sex, weight status, course area/field and economic stratum. A hypothetical causal structural model was elaborated to investigate the impact of sample characteristics on physical and psychological lifestyles. This model was evaluated considering the global fit to the data (χ^2^/df, CFI, TLI and RMSEA) and the hypothetical causal trajectories (β) (α = 5%).

**Results:**

A sample of 1,303 students was used. The mean age was 20.9 (standard deviation = 2.8) years, 66.8% of participants were females, 63.4% had weights in the normal range and 73.7% were students of the social and exact sciences. The PEVI data showed an adequate fit for both the first- (χ^2^/df = 2.03, CFI = 0.98; TLI = 0.97; RMSEA = 0.04) and second-order (χ^2^/df = 2.25; CFI = 0.97; TLI = 0.97; RMSEA = 0.04) models. There was a higher prevalence of unfavorable physical and psychological lifestyle choices among females, among underweight and obese individuals, in older students and in those with lower economic strata. Moreover, negative behaviors in physical lifestyle were more prevalent in students from human/social/exact sciences and worse psychological lifestyle was observed among health sciences students. These results were confirmed by a structural model.

**Conclusion:**

The PEVI data presented validity and reliability. Negative lifestyle choices had high prevalence among students. Moreover, individual characteristics had different impact on physical and psychological lifestyle choices.

## Introduction

Lifestyle can be defined as a set of choices and actions reflecting one’s overall beliefs, values and attitudes towards life (Nahas, Barros & Francalacci, 2000; WHO, 1998). These actions have a personal component that along with the socio-cultural and environmental factors represent the quality of life construct, which is closely related to an individual’s health (Nahas, Barros & Francalacci, 2000; Añez, Reis & Petroski, 2008). According to Nahas, Barros and Francalacci (Nahas, Barros & Francalacci, 2000), this proposed approach to lifestyle is consistent with a holistic view of general health, wherein it results from the successful integration of physical, social, spiritual, emotional and intellectual factors, rather than the mere absence of infirmity (WHO, 1998). Studies of the influence of lifestyle on people’s health has shown that having healthy habits prevent some illnesses that can be triggered by physical inactivity and poor nutrition, mostly chronic and non-communicable diseases (e.g., coronary heart disease and diabetes) (WHO, 1998; Rahati et al., 2014; Slavícek et al., 2008; WHO, 1999). Given the worldwide increasing prevalence of these diseases, research into lifestyle choices is relevant both in the personal and collective contexts since such choices can have beneficial or harmful effects on individuals’ health.

Studies (Nahas, Barros & Francalacci, 2000; Aceijas et al., 2016; Hernandez et al., 2007) indicate that lifestyle has been typically studied from five root components: diet, exercise, preventive behaviors, interpersonal relationships and emotional control. Historically, nutrition, physical activity, and stress management have formed a triad in lifestyle analyses. However, Nahas, Barros & Francalacci (2000) suggested (considering the Brazilian context) that these components alone were insufficient to determine a healthy life, and preventive behaviors and interpersonal relationships were added to their research (Nahas, Barros & Francalacci, 2000), thus moving towards the aforementioned holistic approach in health contexts.

Since lifestyle research probes into individual choices that are not directly measurable, its assessment is usually accomplished through the application of psychometric instruments. The “*FANTASTIC*” (Wilson, Nielsen & Ciliska, 1984), the “*Questionário Saúde na Boa*” (Nahas et al., 2007) and the “*Pentáculo do Bem-Estar*” (also called Individual Lifestyle Profile (“*Perfil do Estilo de Vida Individual*”—PEVI)) (Nahas, Barros & Francalacci, 2000) are some of the instruments designed to evaluate lifestyle choices of individuals. For this study, the PEVI was selected as it was developed in a Brazilian context and it is free of charge.

Although the PEVI (which has a five-pointed star as a symbol) has been employed in lifestyle studies, the instrument was originally developed with educational and clinical awareness purposes (Nahas, Barros & Francalacci, 2000; Both et al., 2008). In addition, studies aimed at analyzing PEVI’s psychometric properties are scarce (Hernandez et al., 2007; Both et al., 2008). To verify whether the instrument does measure the intended concepts, its validity and reliability must be evaluated.

The PEVI has already been used in various contexts, such as among physical education professors (Both et al., 2008, 2010; Lemos, Nascimento & Borgatto, 2007) and university/college (Coelho & Santos, 2006; Santos & Alves, 2009) students. The interest in the study of lifestyles among college-age students (Aceijas et al., 2016; Santos & Alves, 2009; Lee & Loke, 2005; Nelson et al., 2008; Sepulveda, Carrobles & Gandarilhas, 2008) is related to the development period in which they are in (end of adolescence and early adulthood), called “emerging adulthood” (Nelson et al., 2008; Badger, Quatromoni & Morrell, 2019). At that stage, individuals typically begin to explore their own identity and establish autonomy (Nelson et al., 2008), which is reflected in the adoption of new behaviors and lifestyle choices that can be maintained in the long term (Aceijas et al., 2016; Lee & Loke, 2005). Thus, in order to promote healthy behaviors and minimize health risks in the population, lifestyle research among university students has received considerable attention. Studies using the PEVI (Coelho & Santos, 2006; Santos & Alves, 2009) among university students reported significant prevalence of unfavorable behaviors, especially related to nutrition (71.5%; (95% Confidence Interval (CI_95%_): [71.3–71.7])) and physical activity (62.8% (CI_95%_: [62.6–63.0])), which reinforces the concerns regarding the long term effects in the population.

Characteristics such as sex and weight status can also contribute to lifestyle choices (Coelho & Santos, 2006; Denton, Prus & Walters, 2004; Peixoto, Benício & Jardim, 2007; Von Bothmer & Fridlund, 2005). According to Denton, Prus & Walters (2004), clear distinctions exist between males and females regarding lifestyle, affecting their vulnerabilities differently. Weight status (Peixoto, Benício & Jardim, 2007) also has been found to reflect the adopted lifestyle, since it is directly affected by eating habits, physical exercise and emotional control (Nelson et al., 2008; Badger, Quatromoni & Morrell, 2019; Peixoto, Benício & Jardim, 2007).

Several studies on health-related issues select samples from the health sciences student population (Badger, Quatromoni & Morrell, 2019; Melnyk et al., 2016; El-Kassas & Ziade, 2016). As future health professionals, they are seen as a model in such issues (Melnyk et al., 2016). However, students of health areas in general have a greater awareness about the importance of a healthy lifestyle as a protective factor against illnesses (Can et al., 2008), which might not be the case for students of other courses/fields (Aceijas et al., 2016). Therefore, the evaluation of the lifestyle of students from different areas (Can et al., 2008; Sajwani et al., 2009) can identify specific vulnerabilities and difficulties and help in the development of targeted strategies to improve lifestyle choices.

The objective of the present study was to estimate the PEVI’s psychometric properties when applied to a sample of Brazilian university students, identify the prevalence of each lifestyle component according to age, sex, weight status, course area/field and economic stratum and estimate the contribution of these characteristics on physical and psychological lifestyles.

## Materials and Methods

### Study design and participants recruitment

This was a cross-sectional observational study with a non-probabilistic convenience sample. Students between the ages of 18 and 40 years enrolled in undergraduate courses at a Brazilian public university were invited to participate.

A minimum sample size was estimated according to Hair et al. (2005) of 5–10 individuals per parameter/item of models to be estimated. Considering the 35 parameters of the first-order PEVI model and 39 parameters of the second-order model and considering a 20% loss rate (from missing data), the minimum required sample was of 244–488 participants. The total sample was divided randomly in two groups using the function “select cases” in the SPSS software (v.22, IBM, Armonk, NY, USA).

Data related to sex, age, university course area/field and the time of day classes are held (morning, evening, night or full-time), weight, height and economic stratum were also collected. The participants’ body mass index (BMI) was estimated from their self-reported weight and height and cross-referenced with the World Health Organization (WHO, 2000) proposal of anthropometric weight status. According to Campos et al. (2018), self-reported anthropometric measurements are highly associated with measured weight and height. The Brazilian Economic Classification Criteria was used to categorize economic stratum (Brazilian Association of Research Companies (ABEP), 2019).

Data collection was performed in classrooms during normal class, after scheduling and authorization by the class professor. Before filling out the instrument, students were informed of the purpose of the research and made aware that participation was voluntary and anonymous. The students who agreed to participate signed the Informed Consent Form, and filled out demographic data and PEVI questionnaires. The study was approved by the Ethics Committee for Human Research of the School of Pharmaceutical Sciences of UNESP at the Araraquara campus (CAAE: 63553516.4.0000.5426).

### PEVI questionnaire

The PEVI was originally developed in Portuguese by Nahas, Barros and Francalacci (Nahas, Barros & Francalacci, 2000) to evaluate individual and collective lifestyle choices. It features 15 items, evenly divided into five factors addressing Nutrition (items 1–3), Physical Activity (items 4–6), Preventive Behaviors (items 7–9), Social Relationships (items 10–12), and Stress Management (items 13–15). The PEVI contains a four-point response scale with responses ranging from 0 (“Never—absolutely not part of your lifestyle”) to 3 (“Always—absolutely part of your lifestyle”).

The overall score is calculated by averaging the responses given to the items in each factor. Studies have evaluated the prevalence of adequate and inappropriate behaviors for each factor based on the scores (per item and global) (Coelho & Santos, 2006; Santos & Alves, 2009; Badger, Quatromoni & Morrell, 2019). Nahas, Barros and Francalacci (Nahas, Barros & Francalacci, 2000) suggest that scores 0 and 1 indicate unfavorable behaviors and scores 2 and 3 reflect more favorable lifestyle choices. However, in epidemiological contexts, scores in the middle range of the scale do not have a clear indication. Some authors (Both et al., 2010; Lemos, Nascimento & Borgatto, 2007; Moreira et al., 2010) have, therefore, proposed an intermediate category to indicate an amenable lifestyle.

The PEVI’s psychometric properties have been assessed only through exploratory factor analysis (Hernandez et al., 2007; Both et al., 2008). However, considering that the PEVI’s theoretical model was established a priori (Nahas, Barros & Francalacci, 2000), a confirmatory approach should be more appropriate (Anastasi & Urbina, 1997; Silva, Marôco & Campos, 2018).

### Data analysis

Descriptive statistic and shape measures (skewness and kurtosis) were performed. A normal distribution was considered when absolute values of skewness and kurtosis were lower than 3 and 7, respectively (Kline, 2016; Marôco, 2014).

Factorial validity was calculated using confirmatory factor analysis (CFA) with a robust weighted least squares method adjusted for mean and variance (WLSMV). The fit of the model to the data was evaluated using the chi-square per degrees of freedom ratio (χ^2^/df), Comparative Fit Index (CFI), Tucker-Lewis Index (TLI) and Root Mean Square Error of Approximation (RMSEA). The fit was considered adequate when χ^2^/df ≤ 5.0, CFI and TLI ≥ 0.90 and RMSEA ≤ 0.10 (Kline, 2016; Marôco, 2014). Factor loadings (λ) of the items were evaluated and considered adequate if λ ≥ 0.40 (Marôco, 2014). However, for items with λ greater than 0.30, the minimum practical significance of the item was used (Hair et al., 2005). Modification indexes, calculated using the Lagrange Multiplier (LM) method, greater than 11 (*p* < 0.001) were also analyzed (Marôco, 2014). A second-order model was tested based on the grouping into two core factors, a Physical factor (Nutrition, Physical Activity and Preventive Behavior) and a Psychological factor (Social Relationship and Stress Management) which aligns with the conception of general health proposed by Nahas, Barros and Francalacci (Nahas, Barros & Francalacci, 2000).

Convergent validity was assessed by the average variance extracted (AVE) and considered appropriate if ≥0.50 (Fornell & Larcker, 1981). Discriminant validity was performed based on correlation analysis between factors and was classified as appropriate if AVE_*i*_ and AVE_*j*_ ≥ square of the correlation between factors *i* and *j* (*r*_*ij*_^2^) (Fornell & Larcker, 1981).

The reliability of the instrument was estimated from the Composite Reliability (ω) (Fornell & Larcker, 1981) and the ordinal alpha coefficient (α). Values of ω and α ≥ 0.70 were indicative of adequate reliability (Marôco, 2014).

After fitting the model to the data, a second sample was used to verify whether the proposed model for the first sample (Test Sample: *n* = 635) was applicable to an independent sample of the same population (Validation Sample: *n* = 668) using invariance analysis (Kaplan & Saccuzzo, 2012), as proposed by Wu and Estabrook (Wu & Estabrook, 2016). The factorial invariance models of the samples were estimated from multigroup analysis with the CFI difference (ΔCFI) for a series of nested models (Configural (M0), thresholds (M1), factor loadings (M2), regressions (M3), means (M4) and residuals (M5)). Invariance was acceptable when the reduction in CFI (ΔCFI) was less than 0.01. The above analyses were performed using the R software (R Core Team, 2018, v. 3.5.0) with the “lavaan” (Lavaan, 2012) (version 0.6-4) and “semTools” (Jorgensen et al., 2018) (version 0.5-1) packages.

After confirming the PEVI’s validity and the reliability to the sample, the mean scores of the Nutrition, Physical Activity, Preventive Behavior, Social Relationship and Stress Management dimensions were calculated. The mean scores of the Physical and Psychological Lifestyle dimensions (for the second-order model) were also calculated. Based on scores, subjects were grouped according to the lifestyle division of the original proposal (Nahas, Barros & Francalacci, 2000) into favorable choices (positive valence—scores equal or greater than 2) and unfavorable choices (negative valence—scores equal or lower than 1). Scores >1 and <2 were considered having “intermediate choices”. Prevalence was calculated per point and 95% exact confidence intervals (CI_95%_) for each PEVI component factor, as well as for the second-order model, according to sex, weight status, course area/field, age and economic stratum. It should be noted that for age range, prevalence of the lifestyle choices of individuals with 30 years or more was not presented in results because the sample size was small (*n* = 25).

To investigate the impact of sample characteristics on physical and psychological lifestyles, a hypothetical causal structural model was constructed using structural equation modeling. In this model, the physical and psychological lifestyles components were considered dependent variables and sample characteristics were inserted as independent variables in the model. The models were analyzed in two stages. In the first stage, the global fit to the data was verified using the reference values χ^2^/df ≤ 5.00, CFI and TLI ≥ 0.90 and RMSEA ≤ 0.10 (Kline, 2016; Marôco, 2014). In the second stage, the hypothetical causal trajectories (β) were estimated and tested using the *z*-test. For decision making, a significance level of 5% (two-sided) was adopted. When necessary, the model was refined by stepwise regression using the backward elimination method considering non-statistical significance of some trajectories (*p* > 0.05).

## Results

A total of 1,357 students agreed to participate, but only 1,303 fully completed the PEVI (response rate = 96.0%) and were included in the final sample.

The mean age of the participants was 20.9 years (SD = 2.8; minimum = 18.0; 1st quartile = 19.0; median = 20.0; 3rd quartile = 22.0 and maximum = 39.0) and 66.8% were females. The mean BMI was 23.3 kg/m^2^ (SD = 4.1; minimum = 16.0; 1st quartile = 20.5; median = 22.7; 3rd quartile = 25.5 and maximum = 56.5). Additional demographic information of the sample is presented in Table 1.

**Table 1:** Characterization of the test and validation samples.

Feature	Test sample (*n* = 635)	Validation sample (*n* = 668)
	*n* (%)	*n* (%)
Course/field		
Humanities and social sciences	415 (65.4)	443 (66.3)
Exact sciences	54 (8.5)	49 (7.3)
Life and health sciences	166 (26.1)	176 (26.4)
Course year		
First	240 (38.2)	238 (35.7)
Second	164 (26.1)	172 (25.8)
Third	103 (16.4)	121 (18.1)
Fourth	93 (14.8)	106 (15.9)
Fifth	28 (4.5)	30 (4.5)
School/class schedule		
Morning	104 (17.2)	101 (16.2)
Evening	74 (12.2)	104 (16.6)
Night	213 (35.2)	214 (34.2)
Full-time	214 (35.4)	206 (33.0)
Economic stratum (estimated average monthly household income)^#^		
A ($6,069.91)	168 (27.3)	161 (25.2)
B ($2,009.59)	344 (56.0)	373 (58.5)
C ($574.12)	101 (16.4)	100 (15.7)
D and E ($170.98)	2 (0.3)	4 (0.6)
Anthropometric nutritional status (BMI)		
Underweight (<18.5 kg/m^2^)	59 (9.4)	50 (7.6)
Normal range (⊢18.5–25.0 kg/m^2^)	396 (63.3)	417 (63.6)
Preobese (⊢25.0–30.0 kg/m^2^)	141 (22.5)	132 (20.1)
Obesity (≥ 30.0 kg/m^2^)	30 (4.8)	57 (8.7)

**Note:**

# Brazilian economic classification criteria 2019. Brazilian Reals (BRL) were converted into American dollars (exchange rate in November 2019—1 dollar = 4.21 BRL—available in https://www.bcb.gov.br).

Most participants were enrolled in humanities and social sciences courses, in the first and second years of the course (62.8%), attended classes during the day (morning/afternoon/full-time periods—65.3%), belonged to economic strata A or B (83.5%) (i.e., respectively people with high or average purchasing power), and had normal weight (63.4%).

The descriptive statistics of participants’ answers to the PEVI’s items in the test and validation samples are shown in Table 2.

**Table 2:** Descriptive statistics on the answers given to the items of the Individual Lifestyle Profile—PEVI instrument by the participants of the two samples used (Test *n* = 635 and Validation *n* = 668).

Item	Test/validation				
	Mean	Median	SD	Skewness	Kurtosis
(1) Your daily diet includes at least five servings of fruit and vegetables	0.93/0.96	1.00/1.00	0.81/0.86	0.60/0.65	−0.15/−0.21
(2) You avoid eating fatty foods (fatty meats, fried foods) and sweets	1.06/1.04	1.00/1.00	0.83/0.88	0.47/0.50	−0.30/−0.47
(3) You have 4–5 varied meals a day, including a full breakfast	1.27/1.28	1.00/1.00	1.02/1.05	0.38/0.31	−0.95/−1.10
(4) You perform at least 30 min of moderate/intense physical activity on a continuous or cumulative basis, 5 or more days a week	0.87/0.91	0.00/0.00	1.09/1.10	0.92/0.81	−0.60/−0.79
(5) At least twice a week you perform exercises involving muscle strength and stretching	1.21/1.23	1.00/1.00	1.23/1.22	0.39/0.36	−1.47/−1.48
(6) In your daily life, you walk or cycle as a means of transport and preferably use the stairs instead of the elevator	1.37/1.37	1.00/1.00	1.15/1.13	0.20/0.18	−1.40/−1.36
(7) You know your blood pressure, your cholesterol levels and try to manage them	1.11/1.12	1.00/1.00	1.12/1.12	0.47/0.49	−1.20/−1.19
(8) You do not smoke, drink alcohol, or drink alcohol in moderation (less than two servings a day)^*^	1.81/1.79	2.00/2.00	1.23/1.24	−0.38/−0.35	−1.50/−1.53
(9) You always wear seat belts and, if you drive, do so while respecting traffic rules, never drinking alcohol if you drive	2.61/2.54	3.00/3.00	0.73/0.81	−1.95/−1.70	3.25/1.94
(10) You seek to cultivate friendships and are satisfied with your relationships	2.33/2.25	3.00/2.00	0.79/0.80	−0.91/−0.82	−0.01/−0.02
(11) Leisure includes meeting with friends, group sports, memberships	1.84/1.84	2.00/2.00	0.94/0.95	−0.29/−0.28	−0.90/−0.96
(12) You seek to be active in your community, feeling useful in your social environment	1.55/1.50	2.00/1.00	0.99/0.99	0.01/0.09	−1.03/−1.04
(13) You set aside time (at least 5 min) every day to relax	1.99/1.90	2.00/2.00	1.05/1.09	−0.58/−0.45	−0.96/−1.18
(14) You keep a discussion unchanged, even when upset	1.47/1.48	1.00/1.00	0.94/0.92	0.01/0.02	−0.89/−0.82
(15) You balance work time with leisure time	1.39/1.35	1.00/1.00	0.89/0.93	0.17/0.27	−0.70/−0.75

**Note:**

* 1 serving, one can of beer or draft (350 ml), one glass of wine (125 ml) or one serving of spirits (30 ml). SD, standard deviation.

No significant deviation from the normal distribution was observed for any of the PEVI items, underscoring the appropriate psychometric sensitivity of the items.

The PEVI model fitted to the data, considering the confirmatory analysis of the “Test” and “Validation” samples. Moreover, strict invariance was found according to the CFI test (ΔCFI) between independent samples. These results are presented in Table 3.

**Table 3:** Measures of construct validity, reliability and factorial invariance of Individual Lifestyle Profile (PEVI) applied among university students.

Sample	Model	Construct validity								Reliability	
		λ/β	χ^2^/df	CFI	TLI	RMSEA	EI	*r* factors	AVE	ω	α
Test *n* = 635	Original complete	0.317–0.913	2.03	0.98	0.97	0.04	–	0.16–0.60	0.24–0.57	0.45–0.78	0.48–0.75
	Refined 1 (RM1)	0.253–0.915	1.69	0.99	0.98	0.03	8	0.22–0.60	0.15–0.57	0.24–0.78	0.22–0.75
	Original complete SOM	0.422–0.780	2.25	0.97	0.97	0.04	–	0.57	0.24–0.57	0.45–0.78	0.48–0.75
Validation *n* = 668	Original complete	0.278–0.897	2.43	0.98	0.97	0.05	–	0.07–0.64	0.27–0.58	0.47–0.80	0.48–0.77
Test × Validation	Original complete SOM	ΔCFI_M1–M0_ = −0.001; ΔCFI_M2–M1_ = 0.003; ΔCFI_M3–M2_ =< 0.001; ΔCFI_M4–M3_ = 0.004; ΔCFI_M5–M4_ = −0.001									

**Note:**

RM, refined model; SOM, second-order model; λ, items factor loadings; β, hypothetical causal paths of SOM; χ^2^/df, chi-square per degrees of freedom ratio; CFI, comparative fit index; TLI, tucker-lewis index; RMSEA, root mean square error of approximation; EI, excluded items; *r* factors, pearson correlation between factors; AVE, average variance extracted; ω, composite reliability; α, ordinal alpha coefficient; ΔCFI, CFI difference; M0, configural model; M1, *thresholds* model; M2, factor weights/loadings model; M3, regressions model; M4, means model; M5, residuals model.

**Figure 1:**
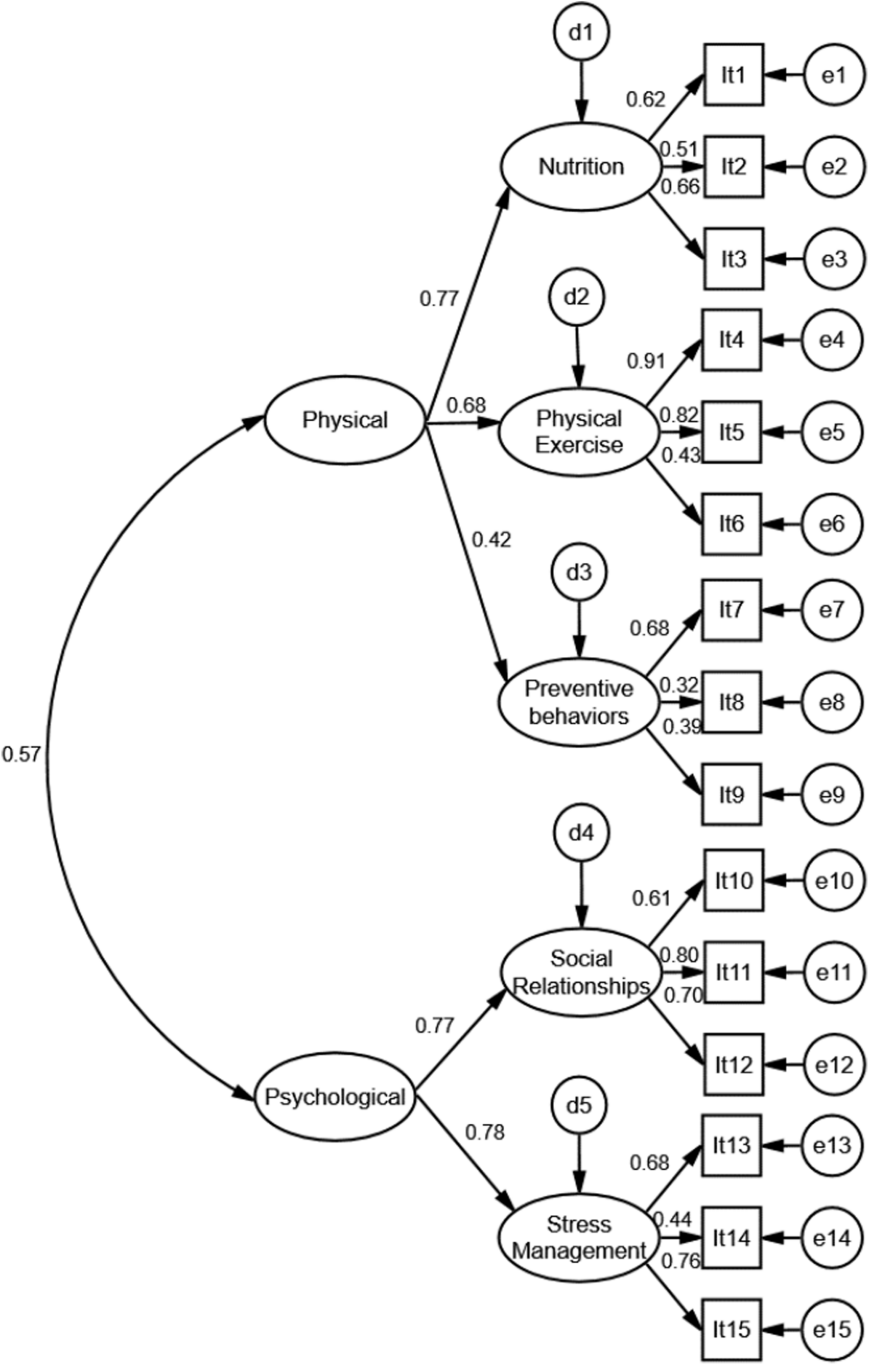
Second-order model of the PEVI instrument (Individual Lifestyle Profile) fitted to the sample of college students.

The original PEVI model presented an overall adequate fit to the sample, however, convergent validity and reliability were not optimal. Model refinement was performed and the items with low factor loading were deleted. After refinement (removal of item eight), validity and reliability estimates of the Preventive Behaviors factor were reduced. Considering that the fit of the overall model to the data was adequate and that the refinement did not improve convergent and discriminant validity and reliability estimates, the original theoretical proposal by Nahas, Barros and Francalacci (Nahas, Barros & Francalacci, 2000) was maintained. The second order model (SOM) also presented adequate fit (Fig. 1).

The total prevalence of individuals grouped according to negative, intermediate and positive options of the first and second-order lifestyle models and according to sex, course area/field, weight status, age and economic stratum is shown in Tables 4–6.

**Table 4:** Distribution of individuals in lifestyle components according to sex and course/field.

PEVI factor	Classification	Sex				Course/field					
		Male (*n* = 433)		Female (*n* = 870)		Human/social + exact sciences (*n* = 961)		Health sciences (*n* = 342)		Total sample	
		*n* (%)	CI_95%_	*n* (%)	CI_95%_	*n* (%)	CI_95%_	*n* (%)	CI_95%_	*n* (%)	CI_95%_
Nutrition	Negative	272 (62.9)	[62.7–63.1]	494 (56.8)	[56.7–56.9]	573 (59.6)	[59.5–59.7]	193 (56.4)	[56.1–56.7]	766 (58.8)	[58.7–58.9]
	Intermediate	95 (21.9)	[21.7–22.1]	243 (27.9)	[27.8–28.0]	234 (24.3)	[24.2–24.4]	104 (30.4)	[30.1–30.7]	338 (25.9)	[25.8–26.0]
	Positive	66 (15.2)	[15.0–15.4]	133 (15.3)	[15.2–15.4]	154 (16.1)	[16.0–16.2]	45 (13.2)	[13.0–13.4]	199 (15.3)	[15.2–15.4]
Physical activity	Negative	173 (40.0)	[39.8–40.2]	535 (61.5)	[61.4–61.6]	497 (51.7)	[51.6–51.8]	211 (61.7)	[61.4–62.0]	708 (54.3)	[54.2–54.4]
	Intermediate	98 (22.6)	[22.4–22.8]	158 (18.2)	[18.1–18.3]	187 (19.5)	[19.4–19.6]	69 (20.2)	[20.0–20.4]	256 (19.7)	[19.6–19.8]
	Positive	162 (37.4)	[37.2–37.6]	177 (20.3)	[20.2–20.4]	277 (28.8)	[28.7–28.9]	62 (18.1)	[17.9–18.3]	339 (26.0)	[25.9–26.1]
Preventive behavior	Negative	114 (26.3)	[26.1–26.5]	150 (17.2)	[17.1–17.3]	225 (23.4)	[23.3–23.5]	39 (11.4)	[11.2–11.6]	264 (20.3)	[20.2–20.4]
	Intermediate	132 (30.5)	[30.3–30.7]	199 (22.9)	[22.8–23.0]	248 (25.8)	[25.7–25.9]	83 (24.3)	[24.1–24.5]	331 (25.4)	[25.3–25.5]
	Positive	187 (43.2)	[43.0–43.4]	521 (59.9)	[59.8–60.0]	488 (50.8)	[50.7–50.9]	220 (64.3)	[64.0–64.6]	708 (54.3)	[54.2–54.4]
Social relationship	Negative	68 (15.7)	[15.5–15.9]	158 (18.1)	[18.0–18.2]	156 (16.2)	[16.1–16.3]	70 (20.5)	[20.3–20.7]	226 (17.3)	[17.2–17.4]
	Intermediate	118 (27.3)	[27.1–27.5]	245 (28.2)	[28.1–28.3]	272 (28.3)	[28.2–8.4]	91 (26.6)	[26.3–26.9]	363 (27.9)	[27.8–28.0]
	Positive	247 (57.0)	[56.8–57.2]	467 (53.7)	[53.6–53.8]	533 (55.5)	[55.4–55.6]	181 (52.9)	[52.6–53.2]	714 (54.8)	[54.7–54.9]
Stress management	Negative	73 (16.9)	[16.7–17.1]	305 (35.0)	[34.9–35.1]	268 (27.9)	[27.8–28.0]	110 (32.2)	[31.9–32.5]	378 (29.0)	[28.9–29.1]
	Intermediate	129 (29.8)	[29.6–30.0]	287 (33.0)	[32.9–33.1]	285 (29.7)	[29.6–29.8]	131 (38.3)	[38.0–38.6]	416 (31.9)	[31.8–32.0]
	Positive	231 (53.3)	[53.1–53.5]	278 (32.0)	[31.9–32.1]	408 (42.4)	[42.3–42.5]	101 (29.5)	[29.2–29.8]	509 (39.1)	[39.0–39.2]
Physical aspect (nutrition, physical activity and preventive behavior )	Negative	126 (29.1)	[28.9–29.3]	291 (33.4)	[33.3–33.5]	318 (33.1)	[33.0–33.2]	99 (28.9)	[28.6–29.2]	417 (32.0)	[31.9–32.1]
	Intermediate	236 (54.5)	[54.3–54.7]	468 (53.8)	[53.7–53.9]	503 (52.3)	[52.2–52.4]	201 (58.8)	[58.5–59.1]	704 (54.0)	[53.9–54.1]
	Positive	71 (16.4)	[16.2–16.6]	111 (12.8)	[12.7–12.9]	140 (14.6)	[14.5–14.7]	42 (12.3)	[12.1–12.5]	182 (14.0)	[13.9–14.1]
Psychological aspect (social relationship and stress management)	Negative	40 (9.2)	[9.1–9.3]	147 (16.9)	[16.8–17.0]	124 (12.9)	[12.8–13.0]	63 (18.4)	[18.2–18.6]	187 (14.4)	[14.3–14.5]
	Intermediate	183 (42.3)	[42.1–42.5]	412 (47.4)	[47.3–47.5]	434 (45.2)	[45.1–45.3]	161 (47.1)	[46.8–47.4]	595 (45.6)	[45.5–45.7]
	Positive	210 (48.5)	[48.3–48.7]	311 (35.7)	[35.6–35.8]	403 (41.9)	[41.8–42.0]	118 (34.5)	[34.2–34.8]	521 (40.0)	[39.9–40.1]

**Note:**

CI_95%_, 95% confidence interval.

**Table 5:** Distribution of individuals in lifestyle components according to weight status.

PEVI factor	Classification	Weight status							
		Underweight (*n* = 109)		Normal range (*n* = 813)		Preobese (*n* = 273)		Obesity (*n* = 87)	
		*n* (%)	CI_95%_	*n* (%)	CI_95%_	*n* (%)	CI_95%_	*n* (%)	CI_95%_
Nutrition	Negative	69 (63.3)	[62.4–64.2]	466 (57.3)	[57.2–57.4]	161 (59.0)	[58.6–59.4]	55 (63.2)	[62.1–64.3]
	Intermediate	28 (25.7)	[24.9–26.5]	207 (25.5)	[25.4–25.6]	72 (26.3)	[26.0–26.6]	26 (29.9)	[28.8–31.0]
	Positive	12 (11.0)	[10.4–11.6]	140 (17.2)	[17.1–17.3]	40 (14.7)	[14.4–15.0]	6 (6.9)	[6.3–7.5]
Physical activity	Negative	65 (59.6)	[58.7–60.5]	425 (52.3)	[52.2–52.4]	147 (53.9)	[53.5–54.3]	56 (64.4)	[63.3–65.5]
	Intermediate	28 (25.7)	[24.9–26.5]	154 (18.9)	[18.8–19.0]	62 (22.7)	[22.4–23.0]	10 (11.5)	[10.8–12.2]
	Positive	16 (14.7)	[14.1–15.3]	234 (28.8)	[28.7–28.9]	64 (23.4)	[23.1–23.7]	21 (24.1)	[23.1–25.1]
Preventive behaviors	Negative	23 (21.1)	[20.4–21.8]	158 (19.4)	[19.3–19.5]	55 (20.1)	[19.8–20.4]	19 (21.8)	[20.8–22.8]
	Intermediate	24 (22.0)	[21.2–22.8]	203 (25.0)	[24.9–25.1]	74 (27.1)	[26.8–27.4]	25 (28.7)	[27.7–29.7]
	Positive	62 (56.9)	[56.0–57.8]	452 (55.6)	[55.5–55.7]	144 (52.8)	[52.4–53.2]	43 (49.5)	[48.3–50.7]
Social relationship	Negative	24 (22.0)	[21.2–22.8]	129 (15.9)	[15.8–16.0]	41 (15.0)	[14.7–15.3]	24 (27.6)	[26.6–28.6]
	Intermediate	37 (33.9)	[33.0–34.8]	219 (26.9)	[26.8–27.0]	84 (30.8)	[30.5–31.1]	19 (21.8)	[20.8–22.8]
	Positive	48 (44.1)	[43.2–45.0]	465 (57.2)	[57.1–57.3]	148 (54.2)	[53.8–54.6]	44 (50.6)	[49.4–51.8]
Stress management	Negative	38 (34.9)	[34.0–35.8]	228 (28.1)	[28.0–28.2]	76 (27.8)	[27.5–28.1]	33 (37.9)	[36.8–39.0]
	Intermediate	27 (24.8)	[24.0–25.6]	267 (32.8)	[32.7–32.9]	84 (30.8)	[30.5–31.1]	29 (33.4)	[32.3–34.5]
	Positive	44 (40.3)	[39.4–41.2]	318 (39.1)	[39.0–39.2]	113 (41.4)	[41.0–41.8]	25 (28.7)	[27.7–29.7]
Physical aspect (nutrition, physical activity and preventive behavior)	Negative	44 (40.4)	[39.5–41.3]	241 (29.6)	[29.5–29.7]	85 (31.1)	[30.8–31.4]	33 (37.9)	[36.8–39.0]
	Intermediate	51 (46.8)	[45.9–47.7]	447 (55.0)	[54.9–55.1]	155 (56.8)	[56.4–57.2]	46 (52.9)	[51.7–54.1]
	Positive	14 (12.8)	[12.2–13.4]	125 (15.4)	[15.3–15.5]	33 (12.1)	[11.9–12.3]	8 (9.2)	[8.5–9.9]
Psychological aspect (social relationship and stress management)	Negative	21 (19.3)	[18.6–20.0]	106 (13.0)	[12.9–13.1]	37 (13.6)	[13.4–13.8]	19 (21.8)	[20.8–22.8]
	Intermediate	42 (38.5)	[37.6–39.4]	378 (46.5)	[46.4–46.6]	127 (46.5)	[46.1–46.9]	40 (46.0)	[44.8–47.2]
	Positive	46 (42.2)	[41.3–43.1]	329 (40.5)	[40.4–40.6]	109 (39.9)	[39.5–40.3]	28 (32.2)	[31.1–33.3]

**Note:**

CI_95%_, 95% confidence interval.

**Table 6:** Distribution of individuals in lifestyle components according to age range and economic stratum.

PEVI factor	Classification	Age range				Economic stratum					
		Under 20 years (*n* = 467)		From 20 to 29 years (*n* = 809)		Level A (*n* = 329)		Level B (*n* = 717)		Levels C, D and E (*n* = 207)	
		*n* (%)	CI_95%_	*n* (%)	CI_95%_	*n* (%)	CI_95%_	*n* (%)	CI_95%_	*n* (%)	CI_95%_
Nutrition	Negative	275 (58.9)	[58.7–59.1]	480 (59.4)	[59.3–59.5]	185 (56.2)	[55.9–56.5]	422 (58.9)	[58.8–59.0]	130 (62.8)	[62.3–63.3]
	Intermediate	130 (27.8)	[27.6–28.0]	201 (24.8)	[24.7–24.9]	98 (29.8)	[29.5–30.1]	175 (24.4)	[24.3–24.5]	54 (26.1)	[25.7–26.5]
	Positive	62 (13.3)	[13.2–13.4]	128 (15.8)	[15.7–15.9]	46 (14.0)	[13.8–14.2]	120 (16.7)	[16.6–16.8]	23 (11.1)	[10.8–11.4]
Physical activity	Negative	266 (57.0)	[56.8–57.2]	430 (53.2)	[53.1–53.3]	170 (51.7)	[51.4–52.0]	400 (55.8)	[55.7–55.9]	114 (55.1)	[54.6–55.6]
	Intermediate	93 (19.9)	[19.7–20.1]	156 (19.2)	[19.1–19.3]	75 (22.8)	[22.5–23.1]	137 (19.1)	[19.0–19.2]	39 (18.8)	[18.4–19.2]
	Positive	108 (23.1)	[22.9–23.3]	223 (27.6)	[27.5–27.7]	84 (25.5)	[25.2–25.8]	180 (25.1)	[25.0–25.2]	54 (26.1)	[25.7–26.5]
Preventive behavior	Negative	90 (19.3)	[19.1–19.5]	170 (21.0)	[20.9–21.1]	66 (20.0)	[19.8–20.2]	143 (19.9)	[19.8–20.0]	46 (22.2)	[21.8–22.6]
	Intermediate	112 (24.0)	[23.8–24.2]	215 (26.6)	[26.5–26.7]	93 (28.3)	[28.0–28.6]	176 (24.6)	[24.5–24.7]	46 (22.2)	[21.8–22.6]
	Positive	265 (56.7)	[56.5–56.9]	424 (52.4)	[52.3–52.5]	170 (51.7)	[51.4–52.0]	398 (55.5)	[55.4–55.6]	115 (55.6)	[55.1–56.1]
Social relationship	Negative	77 (16.5)	[16.3–16.7]	142 (17.6)	[17.5–17.7]	38 (11.6)	[11.4–11.8]	119 (16.6)	[16.5–16.7]	56 (27.1)	[26.7–27.5]
	Intermediate	137 (29.3)	[29.1–29.5]	220 (27.2)	[27.1–27.3]	82 (24.9)	[24.6–25.2]	213 (29.7)	[29.6–29.8]	58 (28.0)	[27.6–28.4]
	Positive	253 (54.2)	[54.0–54.4]	447 (55.2)	[55.1–55.3]	209 (63.5)	[63.2–63.8]	385 (53.7)	[53.6–53.8]	93 (44.9)	[44.4–45.4]
Stress management	Negative	123 (26.3)	[26.1–26.5]	245 (30.3)	[30.2–30.4]	66 (20.1)	[19.9–20.3]	212 (29.6)	[29.5–29.7]	85 (41.0)	[40.5–41.5]
	Intermediate	158 (33.8)	[33.6–34.0]	249 (30.8)	[30.7–30.9]	103 (31.3)	[31.0–31.6]	238 (33.2)	[33.1–33.3]	61 (29.5)	[29.1–29.9]
	Positive	186 (39.9)	[39.7–40.1]	315 (38.9)	[38.8–39.0]	160 (48.6)	[48.3–48.9]	267 (37.2)	[37.1–37.3]	61 (29.5)	[29.1–29.9]
Physical aspect (nutrition, physical activity and preventive behavior )	Negative	141 (30.2)	[30.0–30.4]	271 (33.5)	[33.4–33.6]	96 (29.2)	[28.9–29.5]	227 (31.7)	[31.6–31.8]	77 (37.2)	[36.7–37.7]
	Intermediate	272 (58.2)	[58.0–58.4]	419 (51.8)	[51.7–51.9]	188 (57.1)	[56.8–57.4]	393 (54.8)	[54.7–54.9]	98 (47.3)	[46.8–47.8]
	Positive	54 (11.6)	[11.5–11.7]	119 (14.7)	[14.6–14.8]	45 (13.7)	[13.5–13.9]	97 (13.5)	[13.4–13.6]	32 (15.5)	[15.2–15.8]
Psychological aspect (social relationship and stress management)	Negative	58 (12.4)	[12.3–12.5]	121 (15.0)	[14.9–15.1]	26 (7.9)	[7.7–8.1]	101 (14.1)	[14.0–14.2]	54 (26.1)	[25.7–26.5]
	Intermediate	223 (47.8)	[47.6–48.0]	361 (44.6)	[44.5–44.7]	136 (41.3)	[41.0–41.6]	345 (48.1)	[48.0–48.2]	89 (43.0)	[42.5–43.5]
	Positive	186 (39.8)	[39.6–40.0]	327 (40.4)	[40.3–40.5]	167 (50.8)	[50.5–51.1]	271 (37.8)	[37.7–37.9]	64 (30.9)	[30.5–31.3]

**Note:**

CI_95%_, 95% confidence interval.

A greater number of individuals grouped in the Negative lifestyle choices was found across the total sample when evaluating the Nutrition and Physical Activity dimensions. In relation to the other aspects, most participants demonstrated favorable lifestyle choices/positive behaviors. When considering the second-order aspects/model, there was a higher prevalence of people with a negative lifestyle for the Physical aspect and positive for the Psychological aspect.

A greater number of females with unfavorable lifestyle choices in Physical Activity, Social Relationships and Stress Management was observed. For males, a higher prevalence of unfavorable lifestyle choices was found in the Nutrition and Preventive Behavior dimensions. For the second-order model, there was a higher prevalence of females with negative lifestyle for the Physical and Psychological aspects compared to males.

Regarding the course area/field, a negative behavior was more prevalent in Nutrition and Preventive Behaviors among humanities, social and exact sciences students. In turn, among health sciences students, a higher prevalence of negative lifestyles related to Physical Activity, Social Relationships and Stress Management was found. For the second-order model, worse Physical lifestyle choices were found among humanities, social and exact sciences students and worse Psychological lifestyles were seen among health sciences students.

The proportion of obese participants with unfavorable lifestyle choices across the Physical Activity, Social Relationships and Stress Management dimensions was significantly higher than participants with non-obese weight status. For the second-order model, a higher prevalence of underweight and obese individuals had a negative lifestyle for the Physical and Psychological aspects, respectively, compared to individuals with normal weight.

Regarding age, a higher proportion of older students had negative lifestyles choices. Furthermore, a higher prevalence of individuals in economic strata C, D and E (people with lower purchasing power than people of A and B strata) also had negative lifestyles.

**Figure 2:**
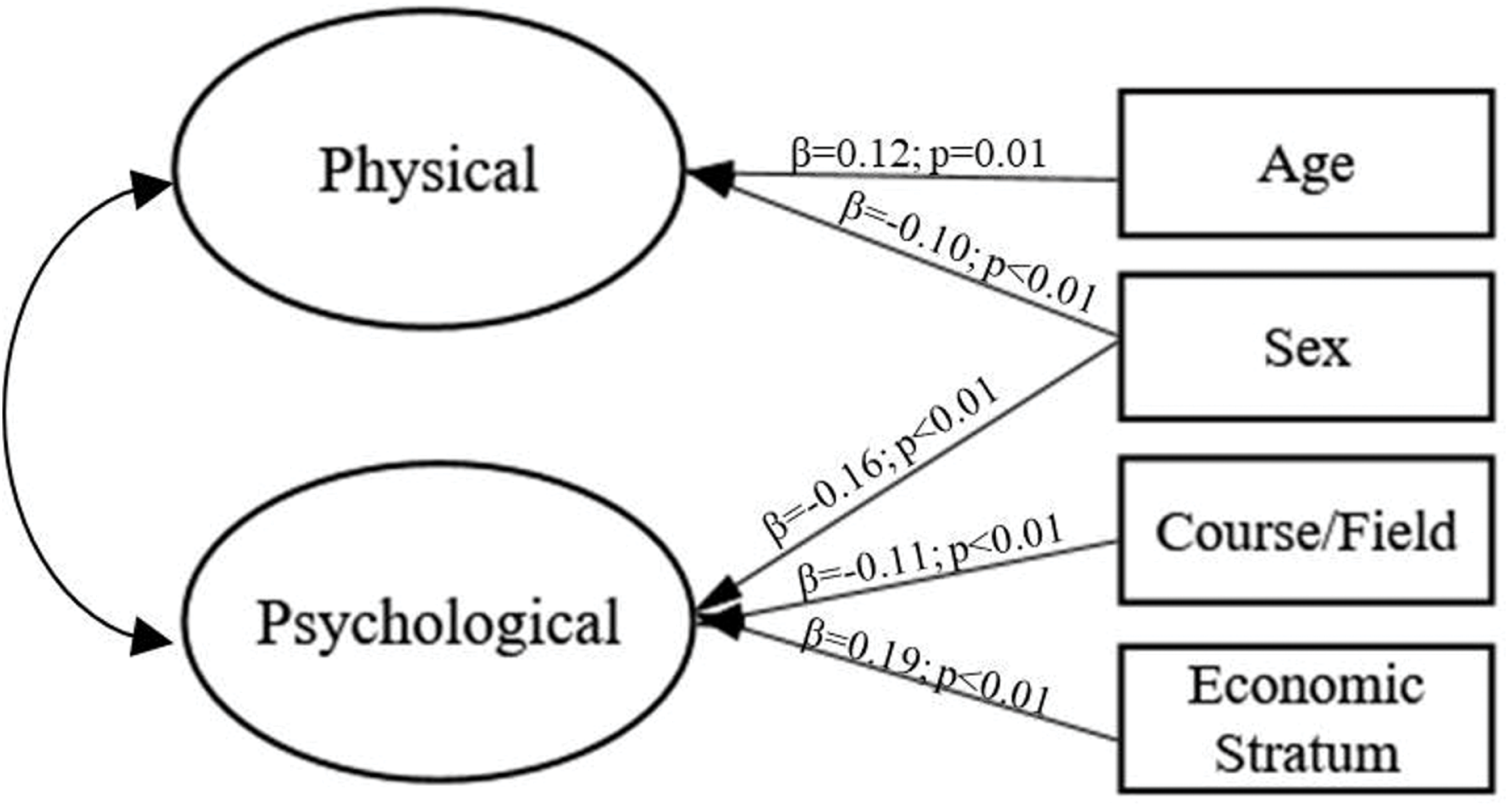
Influence of sample characteristics on physical and psychological lifestyles (hypothetical causal structural model).

The complete model (including the impact of sex, age, weight status, course area/field and economic stratum on physical and psychological lifestyles) fitted marginally to the data (χ^2^/df = 5.09, CFI = 0.88, TLI = 0.92 and RMSEA = 0.06); however, non-significant trajectories (*p* > 0.05) were found. After the stepwise procedure, the model achieved more adequate fit to sample (χ^2^/df = 5.04, CFI = 0.90, TLI = 0.92 and RMSEA = 0.06); regarding χ^2^/df index, it was influenced by sample size, and thus, it cannot be considered without other indexes to accept or reject model’s fit. Sex and age were significant for the physical lifestyle component and sex, course area/field and economic stratum were significant for the psychological component. The representation of the structural model is shown in Fig. 2.

## Discussion

The present study aimed to establish evidence of validity and reliability of the PEVI instrument among college/university students. There was found to be a high prevalence of college students with negative choices for Physical aspects of lifestyle (Nutrition, Physical Activity and Preventive Behavior) and positive choices for Psychological aspects of lifestyle (Social Relationship and Stress Management). There was a higher prevalence of negative choices in both the Physical and Psychological components among females, underweight and obese individuals, in older students and in those with lower economic strata. Moreover, different deficits regarding lifestyle choices were observed according to course area/field.

The theoretical model proposed for the PEVI demonstrated an adequate fit to data. The low convergent validity and reliability observed in some of the instrument’s factors can be justified by the low number of items per factor and the factor loading of the Preventive Behavior component (Hernandez et al., 2007; Both et al., 2008). However, as it did not compromise the model’s fit, the instrument’s original theoretical model was maintained.

Hernandez et al. (2007) and Both et al. (2008) conducted exploratory factor analysis of the PEVI and found different results in terms of the number of factors and in item allocation, which might have been due to the sample characteristics of each study (physical education teachers vs. regular population). Such results suggest that the psychometric properties obtained are related to the data and not to the instrument per se (Marôco, 2014) and, therefore, the instrument’s properties should be assessed whenever it is applied to different samples and in different contexts. Moreover, considering that the PEVI was developed from a theoretical model (Nahas, Barros & Francalacci, 2000), this model should be tested on these samples by confirmatory factor analysis (CFA); exploratory analysis is justified only if the original model was refuted (Anastasi & Urbina, 1997; Silva, Marôco & Campos, 2018) in CFA. As no previous study assessed the operationalization of lifestyle concepts using the PEVI among university students, our results have no data to be compared with.

The data obtained showed that the assessment of lifestyle from a hierarchical model is pertinent since the model presented adequate fit to the sample and reflected the original theoretical conception (Nahas, Barros & Francalacci, 2000) of the instrument, which is that lifestyle is closely related to health status and its evaluation is based on physical and emotional/psychological aspects (WHO, 1998).

Students in general presented unfavorable lifestyle choices for the Nutrition and Physical Activity aspects, which might be related to their difficulty in managing the daily university routine (Lee & Loke, 2005), due to high curricular and extracurricular demands (Aceijas et al., 2016; Badger, Quatromoni & Morrell, 2019). Thus, students’ lifestyle can be marked by physical inactivity and unhealthy eating habits (Nelson et al., 2008). On the other hand, there was a high prevalence of individuals with intermediate and positive lifestyles in the Social Relationship and Stress Management aspects. In the study conducted by Santos & Alves (2009), the prevalence of positive behaviors in Social Relationships (69.3%; (CI_95%_ [69.1–69.5])) and Stress Management (54.4%, (CI_95%_ [54.2–54.6])) among college students was also high, which suggests that college students have a good psychological lifestyle. Perhaps the autonomy and enthusiasm related to the integration into the university environment may favor social interactions, exchange of experiences and leisure opportunities. Thus, to promote healthier lifestyles among these young people, professors and health professionals should focus on strategies to improve mainly the Physical dimensions of lifestyle choices.

Similar to our study, the difference in prevalence of males and females with an unfavorable lifestyle in PEVI’s components was also observed in the study carried out by Coelho & Santos (2006). Males and females can have different lifestyles in part because they have different exposures to health-related demands and coping strategies (Denton, Prus & Walters, 2004). Males usually have greater alcohol consumption, smoking habits and unbalanced nutrition (Denton, Prus & Walters, 2004; Von Bothmer & Fridlund, 2005). Among females, studies indicate that biopsychosocial issues, physical inactivity, physical health and body weight issues are significant health factors (Denton, Prus & Walters, 2004; Von Bothmer & Fridlund, 2005). Such distinctions might explain our finding in the prevalence and structural model analysis that males had better physical and psychological lifestyles choices than females. It should be noted that despite the possibility of considering a global aspect of PEVI (i.e., Physical or Psychological factor), the interpretation of each factor for samples is also important to identify specific vulnerabilities of groups. Thus, we suggest that lifestyle characteristics be investigated in a context considering the general and specific aspects in order to provide a more broad assessment of these concepts.

The low weight and obese subgroups had higher prevalence of unfavorable behaviors. Peixoto, Benício & Jardim (2007) and Abdel-Megeid, Abdelkarem & El-Fetouh (2011) observed a link between weight change, poor nutrition and sedentary behaviors in university populations (Peixoto, Benício & Jardim, 2007; Abdel-Megeid, Abdelkarem & El-Fetouh, 2011). There is a clear interaction between physical lifestyle (e.g., diet and exercise habits) and the emotional aspects of body weight control (Badger, Quatromoni & Morrell, 2019). Thus, the adoption of unfavorable behaviors in relation to food consumption and physical exercise, coupled with ineffective coping strategies (Badger, Quatromoni & Morrell, 2019) can lead to significant changes in weight and subsequent damage to health status (Pôrto et al., 2015). Therefore, weight status and the practice of physical exercise were considered relevant towards understanding lifestyles and were taken into account in our study.

The worse physical lifestyle choices among humanities/social and exact sciences students compared to health sciences students can be associated with the health-related content of health sciences courses (Melnyk et al., 2016; El-Kassas & Ziade, 2016; Sajwani et al., 2009). This acquired expertise can motivate health sciences students to adopt more appropriate lifestyles than students from other fields, once they better understand the consequences of poor nutrition and sedentary habits for overall health (Can et al., 2008; Sajwani et al., 2009). In contrast, a higher prevalence of negative behaviors in psychological aspects of lifestyle was observed among health sciences students, a finding confirmed in the structural model. The high academic demand of health sciences courses can impair social relationships and increase stress experiences, and the continuity of such situations can affect the mental and physical health (Badger, Quatromoni & Morrell, 2019; El-Kassas & Ziade, 2016) of the students, which, in turn, may undermine their academic performance (Melnyk et al., 2016), resulting in a vicious cycle of high academic demands, stress and impaired performance. Thus, the assessment of lifestyles in different fields of study identified specific vulnerabilities that allow development of targeted actions in educational environments.

We found that older individuals presented a better physical lifestyle. Nelson et al. (2008) highlight that early adulthood (from 18 to 24 years) can be marked by changes in physical lifestyles, especially in terms of physical activity and eating behaviors, which can be detrimental for health. Moreover, individuals in low economic stratum presented worse psychological lifestyle. According to Molarius et al. (2009), this result is common in other contexts and countries. Individuals with distinct financial resources are under different stress exposure, and poorer individuals face a greater environmental adversity (Dohrenwend, 1990). In addition, health inequalities can be associated with social difference that may result from economic inequalities (Denton, Prus & Walters, 2004). Therefore, our results highlight the importance of promoting strategies for improving psychological lifestyles, especially in students from lower economic strata, to avoid effects on their mental health.

Alvarenga & Koritar (2015) discuss the common belief that maintaining good health conditions is usually associated with restrictions and prohibitions—especially with regards to food (duality between health and pleasure). Given this premise, the hedonic aspects of food are often overlooked (Nahas, Barros & Francalacci, 2000; Alvarenga & Koritar, 2015; Jallinoja, Pajari & Absetz, 2010), which could justify a weak personal engagement to having a balanced diet. Thus, successful and effective strategies promoting healthier lifestyles should feature a biopsychosocial aspect and consider a balance between the individual, their emotions, and the context in which people are integrated (Jallinoja, Pajari & Absetz, 2010; Brito et al., 2019). Moreover, the low involvement in physical exercise may be associated with difficulties with individual motivations (Brand & Cheval, 2019; Ryan et al., 1997). Being sedentary may due to a lack of motivation to engage in physical activity (simply not recognizing the lasting benefits that exercise can promote) or alternately due to viewing such activities as “obligations” (Jallinoja, Pajari & Absetz, 2010; Ryan et al., 1997). Moreover, many current technologies and innovations in the academic environment spare the need of physical effort (Nahas, Barros & Francalacci, 2000; Jallinoja, Pajari & Absetz, 2010; Brand & Cheval, 2019), which also can promote physical inactivity.

The present study does not establish causal relationships since it was a cross-sectional study. However, the presented findings are properly supported by validity and reliability estimates. Moreover, the performed analysis can be useful in the evaluation of lifestyle choices among Brazilian young adults. The generalizability of the results may be hampered by the non-probabilistic sampling. Additionally, we suggest that future studies be conducted in order to incorporate more psychosocial aspects (e.g., social support, affectivities, coping), which can be related with lifestyle choices.

The findings on lifestyle components in different subgroups provided information that may help the establishment of targeted preventive-educational interventions, such as nutritional education, physical activity information, and promotion of health-related preventive behaviors and healthy pleasures.

## Conclusions

The PEVI questionnaire demonstrated validity and reliability in the evaluation of lifestyle among university students. Females, underweight and obese individuals, older students and individuals with lower economic strata had significantly more unfavorable lifestyles both in the physical and psychological aspects. The physical and psychological negative lifestyles also differed according to students’ course area/field. These findings may guide the planning and implementation of health promotion interventions, designed to foster the adoption of healthier lifestyles, considering the inherent characteristics of university students.

## Supplemental Information

10.7717/peerj.9830/supp-1 Supplemental Information 1 R Codes: packages, database and tested models. Each chunk in R file represents one analysis.

10.7717/peerj.9830/supp-2 Supplemental Information 2 Participants’ characteristics and answers given to PEVI. Raw data related to students’ characteristics and answers of PEVI

10.7717/peerj.9830/supp-3 Supplemental Information 3 Empty copy of the Portuguese questionnaire PEVI.

10.7717/peerj.9830/supp-4 Supplemental Information 4 Individual Lifestyle Profile—English Version.

## Additional Information and Declarations

### Data Availability

The following information was supplied regarding data availability: The raw data, code, the questionnaire and an English translation are available in the Supplemental Files.
