# Supplementary material for: Lifestyle choices of Brazilian college students
Source: PeerJ. 2020 Oct 7;8:e9830. doi: 10.7717/peerj.9830 (PMC7547619; doi:10.7717/peerj.9830)
Supplement: Supplemental Information 3 [file peerj-08-9830-s003.pdf]

Questionário de Estilo de Vida individual (PEVI)

Instruções: O ESTILO DE VIDA corresponde ao conjunto de ações habituais que refletem as atitudes e valores das pessoas. Essas ações têm grande influência na saúde geral e na qualidade de vida de todos os indivíduos. **Os itens abaixo representam características do estilo de vida relacionadas ao bem-estar individual. Manifeste-se sobre cada afirmação considerando a escala:**

**Item**	**Absolutamente não faz parte do seu estilo de vida**	**Às vezes corresponde ao seu comportamento**	**Quase sempre verdadeiro no seu comportamento**	**A afirmação é sempre verdadeira no seu dia a dia, faz parte do seu estilo de vida**
**1.** Sua alimentação diária inclui ao menos 5 porções de frutas e verduras.	0	1	2	3
**2.** Você evita ingerir alimentos gordurosos (carnes gordas, frituras) e doces.	0	1	2	3
**3.** Você faz 4 a 5 refeições variadas ao dia, incluindo café da manhã completo.	0	1	2	3
**4.** Você realiza ao menos 30 minutos de atividade físicas moderadas/intensas de forma continua ao acumulada, 5 ou mais dias na semana.	0	1	2	3
**5.** Ao menos duas vezes por semana você realiza exercícios que envolvam força e alongamento muscular.	0	1	2	3
**6.** No seu dia a dia, você caminha ou pedala como meio de transporte e, preferencialmente, usa as escadas ao invés do elevador.	0	1	2	3
**7.** Você conhece sua PRESSÃO ARTERIAL, seus níveis de COLESTEROL e procura controlá-los.	0	1	2	3
**8.** Você NÃO FUMA, não ingere ÁLCOOL OU ingere ÁLCOOL com moderação (menos de 2 doses ao dia). **1 Dose = uma lata de cerveja ou chope (350 ml), uma taça de vinho (125 ml) ou uma dose de destilado (30 ml).**	0	1	2	3
**9.** Você sempre usa cinto de segurança e, se dirige, o faz respeitando as normas de trânsito, nunca ingerindo álcool se vai dirigir.	0	1	2	3
**10.** Você procura cultivar amigos e está satisfeito com os seus relacionamentos.	0	1	2	3
**11.** Seu lazer inclui reuniões com amigos, atividades esportivas em grupo, participações em associações.	0	1	2	3
**12.** Você procura ser ativo em sua comunidade, sentindo-se útil no seu ambiente social.	0	1	2	3
**13.** Você reserva tempo (ao menos 5 minutos) todos os dias para relaxar.	0	1	2	3
**14.** Você mantém uma discussão sem alterar-se, mesmo quando contrariado.	0	1	2	3
**15.** Você equilibra o tempo dedicado ao trabalho com o tempo dedicado ao lazer.	0	1	2	3
