# Supplementary material for: Lifestyle choices of Brazilian college students
Source: PeerJ. 2020 Oct 7;8:e9830. doi: 10.7717/peerj.9830 (PMC7547619; doi:10.7717/peerj.9830)
Supplement: Supplemental Information 4 [file peerj-08-9830-s004.docx]

**Individual Lifestyle Profile (PEVI)**

| **Item** | **Absolutely not part of your lifestyle** | **Sometimes corresponds to your behavior** | **Almost always true in your behavior** | **Absolutely part of your lifestyle** |
| --- | --- | --- | --- | --- |
| **1)** Your daily diet includes at least 5 servings of fruit and vegetables. | 0 | 1 | 2 | 3 |
| **2)** You avoid eating fatty foods (fatty meats, fried foods) and sweets. | 0 | 1 | 2 | 3 |
| **3)** You have 4 to 5 varied meals a day, including a full breakfast. | 0 | 1 | 2 | 3 |
| **4)** You perform at least 30 minutes of moderate/intense physical activity on a continuous or cumulative basis, 5 or more days a week. | 0 | 1 | 2 | 3 |
| **5)** At least twice a week you perform exercises involving muscle strength and stretching. | 0 | 1 | 2 | 3 |
| **6)** In your daily life, you walk or cycle as a means of transport and preferably use the stairs instead of the elevator. | 0 | 1 | 2 | 3 |
| **7)** You know your blood pressure, your cholesterol levels and try to manage them. | 0 | 1 | 2 | 3 |
| **8)** You do not smoke, drink alcohol, or drink alcohol in moderation (less than 2 servings a day)*. | 0 | 1 | 2 | 3 |
| **9)** You always wear seat belts and, if you drive, do so while respecting traffic rules, never drinking alcohol if you drive. | 0 | 1 | 2 | 3 |
| **10)** You seek to cultivate friendships and are satisfied with your relationships. | 0 | 1 | 2 | 3 |
| **11)** Leisure includes meeting with friends, group sports, memberships. | 0 | 1 | 2 | 3 |
| **12)** You seek to be active in your community, feeling useful in your social environment. | 0 | 1 | 2 | 3 |
| **13)** You set aside time (at least 5 minutes) every day to relax. | 0 | 1 | 2 | 3 |
| **14)** You keep a discussion unchanged, even when upset. | 0 | 1 | 2 | 3 |
| **15)** You balance work time with leisure time. | 0 | 1 | 2 | 3 |

**Instructions:** Lifestyle can be defined as a set of choices and actions reflecting one's overall beliefs, values, and attitudes towards life. The items below represent lifestyle features that are related to individual well-being. Please choose the option according to provided response scale.
